# Supplementary figures and images for: Macrophage invasion into the Drosophila brain requires JAK/STAT-dependent MMP activation in the blood–brain barrier
Source: PLoS Biol. 2025 Feb 20;23(2):e3003035. doi: 10.1371/journal.pbio.3003035 (PMC11908702; doi:10.1371/journal.pbio.3003035)

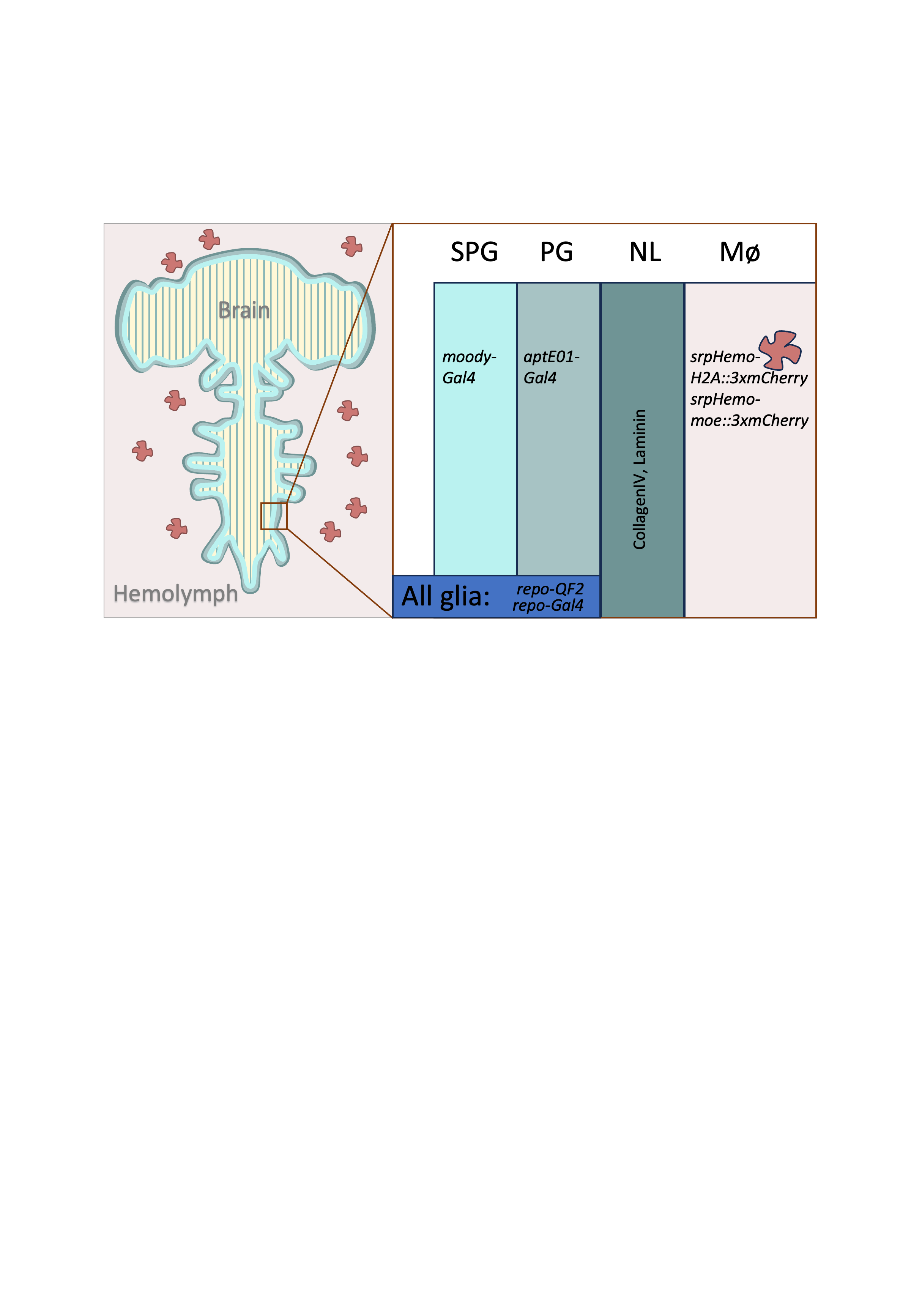

Supplement: S1 Fig — Schematic representation of all main tools used in this study. repo-Gal4 and repo-QF2 direct expression in all glial cells. moody-Gal4 directs expression in the subperineurial glia (SPG), aptE01-Gal4 directs expression in the perineurial glia (PG). The neural lamella (NL) can be labelled using a GFP protein-trap insertion in the collagen IV gene or by staining using anti-Laminin-γ. Macrophages (Mø) can be visualized using srpHemo-H2A::3xmCherry or by srpHemo-moe::3xmCherry. (TIFF) [file pbio.3003035.s001.tiff]

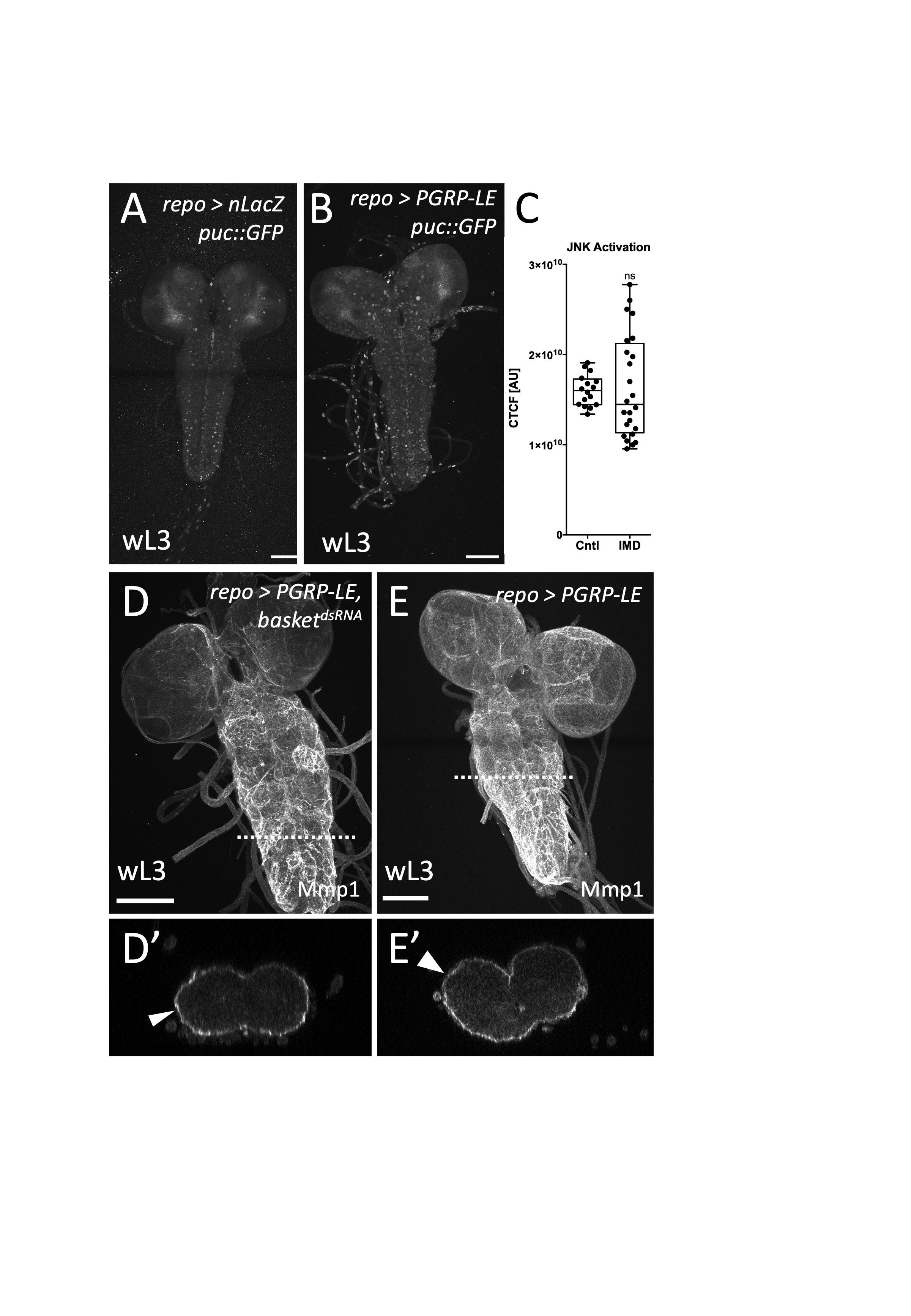

Supplement: S2 Fig — (A, B) Third instar larval brains of control (A) and immunity induction (B) expressing the JNK reporter construct puc::GFP. Scale bar, 100 µm. (C) Measurement of JNK signaling activation in the CNS of third instar larvae with and without panglial immunity induction. No significant difference is detected. Mann–Whitney, P = 0.5543. Control n = 16, IMD n = 24. The corrected total cell fluorescence (CTCF) was calculated in arbitrary units (AU), see Materials and methods for details. For quantification see S8 Data. (D, E) Third instar larval brains with a panglial immunity induction (D) and simultaneous basket downregulation (E) stained for Mmp1. Note the Mmp1 signal at the surface of the CNS (D′, E′, arrowheads). Scale bar, 100 µm. (TIFF) [file pbio.3003035.s002.tiff]

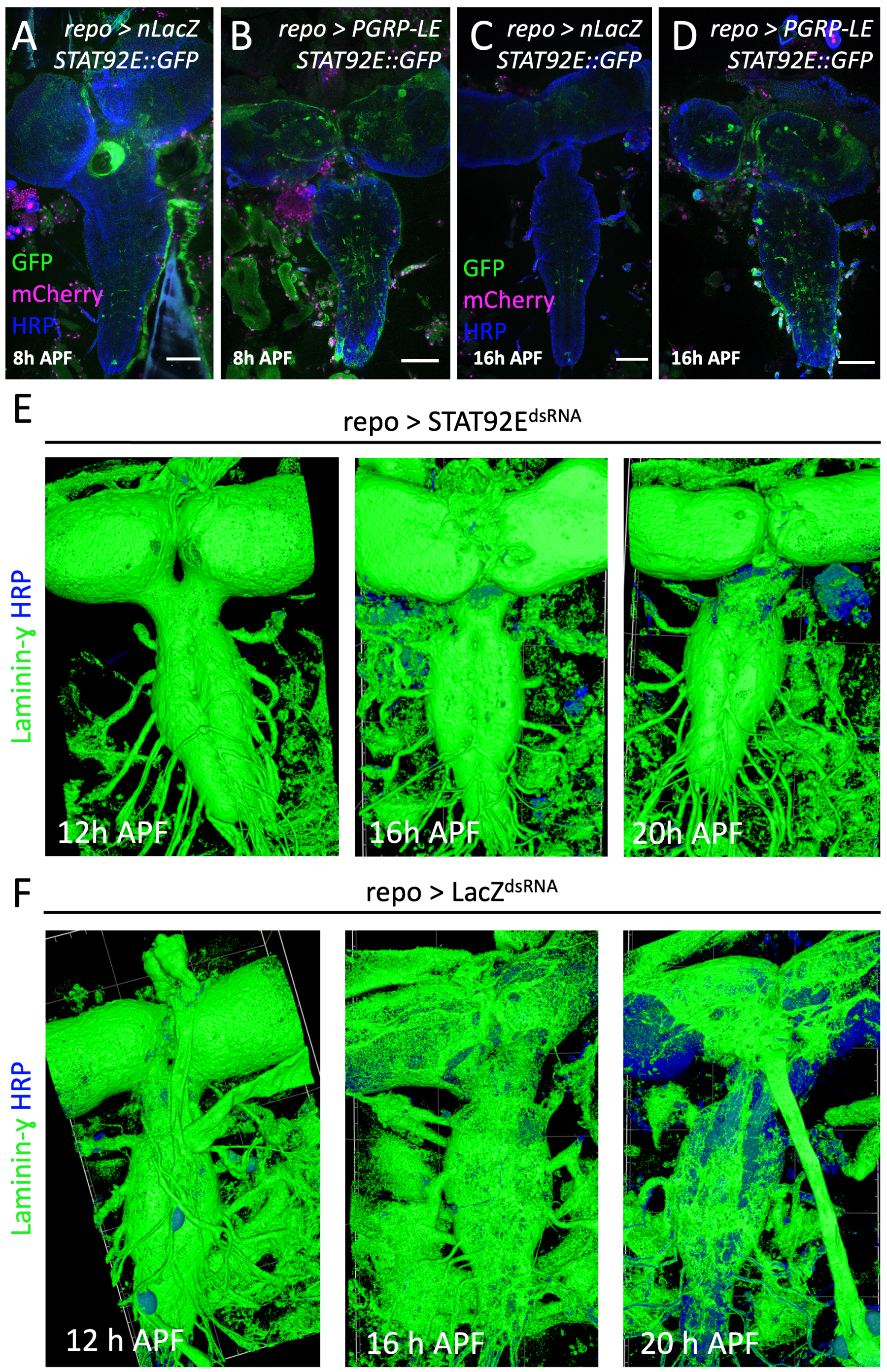

Supplement: S3 Fig — Inhibition of panglial JAK/STAT signaling delays neural lamella remodeling (A–D) Dissected brains of pupae carrying a JAK/STAT signaling reporter. The age and genotypes are indicated. Brains were stained for JAK/STAT signaling activation (green), HRP (blue) to label neuronal membranes and mCherry expression (magenta) directed by the macrophage marker srpHemo-moe::3xmCherry. Note the increased GFP signal upon a panglial immunity induction. Scale bars, 100 µm. (E, F) Pupal brains stained for Laminin-γ (green) and HRP (blue) upon a panglial STAT92E knockdown (E) and controls expressing LacZdsRNA. Neural lamella remodeling is inhibited upon panglial STAT92E knockdown compared to controls. Laminin-γ surfaces were generated with arivis4D. (TIFF) [file pbio.3003035.s003.tiff]

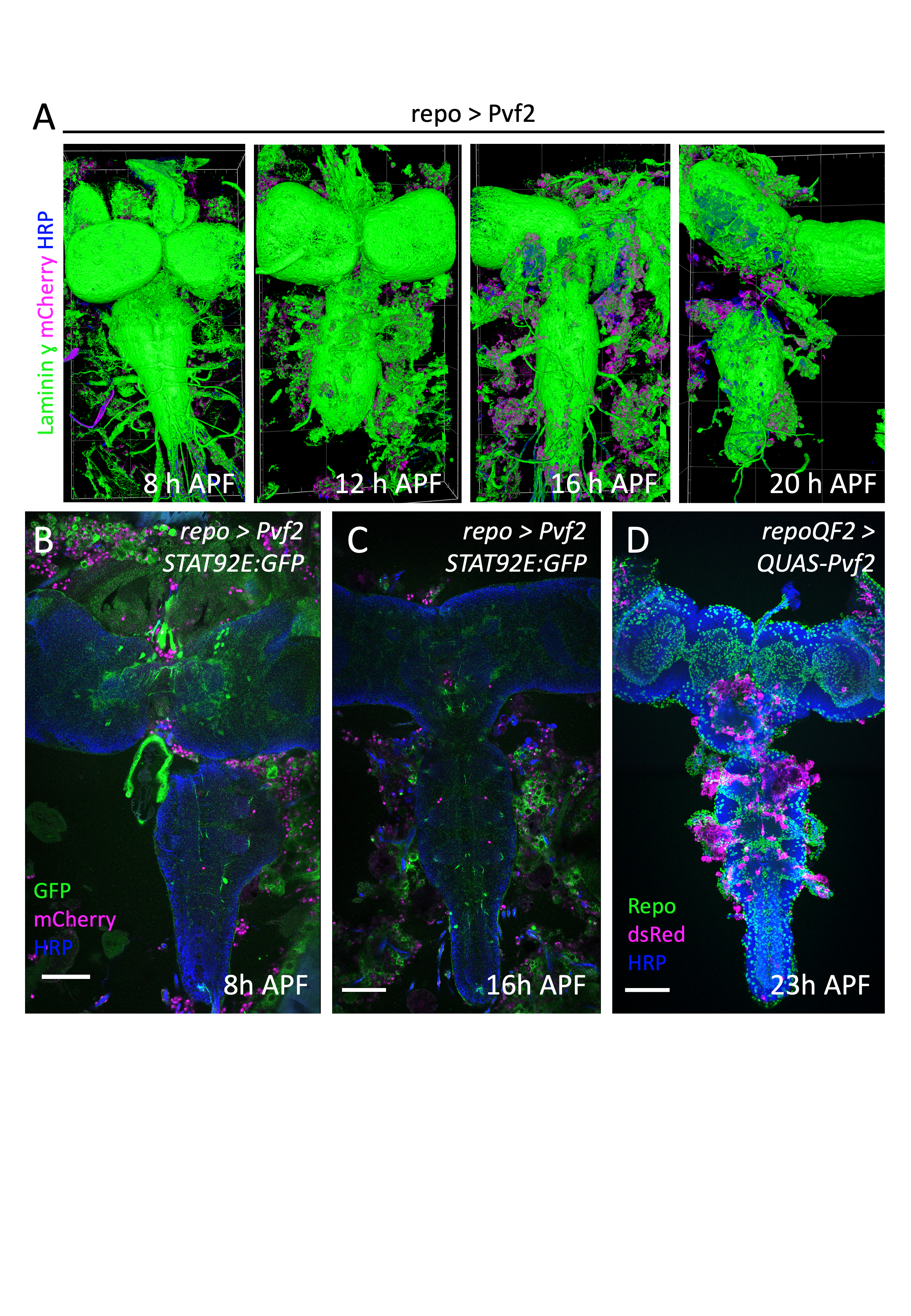

Supplement: S4 Fig — (A) Pupal brains with a panglial Pvf2 expression stained for Laminin-γ (green), HRP (blue) and a macrophage marker (mCherry expression (magenta) directed by srpHemo-moe::3xmCherry). The neural lamella remodeling starts in 20 h APF pupal stages. (B, C) JAK/STAT signaling is not activated in pupal stages upon a panglial Pvf2 expression. Pupal brains expressing Pvf2 and a JAK/STAT signaling reporter of indicated ages stained for STAT92E activation (green), HRP (blue) a macrophage marker (mCherry expression (magenta) directed by srpHemo-moe::3xmCherry). (D) Pupal CNS expressing Pvf2 in all glial cells stained with repo (green) to label glial nuclei, HRP (blue) to label neuronal membranes, and dsRed (magenta) directed by the macrophage marker srpHemo-moe::3xmCherry. Many macrophages are located within the CNS. Scale bars, 100 µm. (TIFF) [file pbio.3003035.s004.tiff]

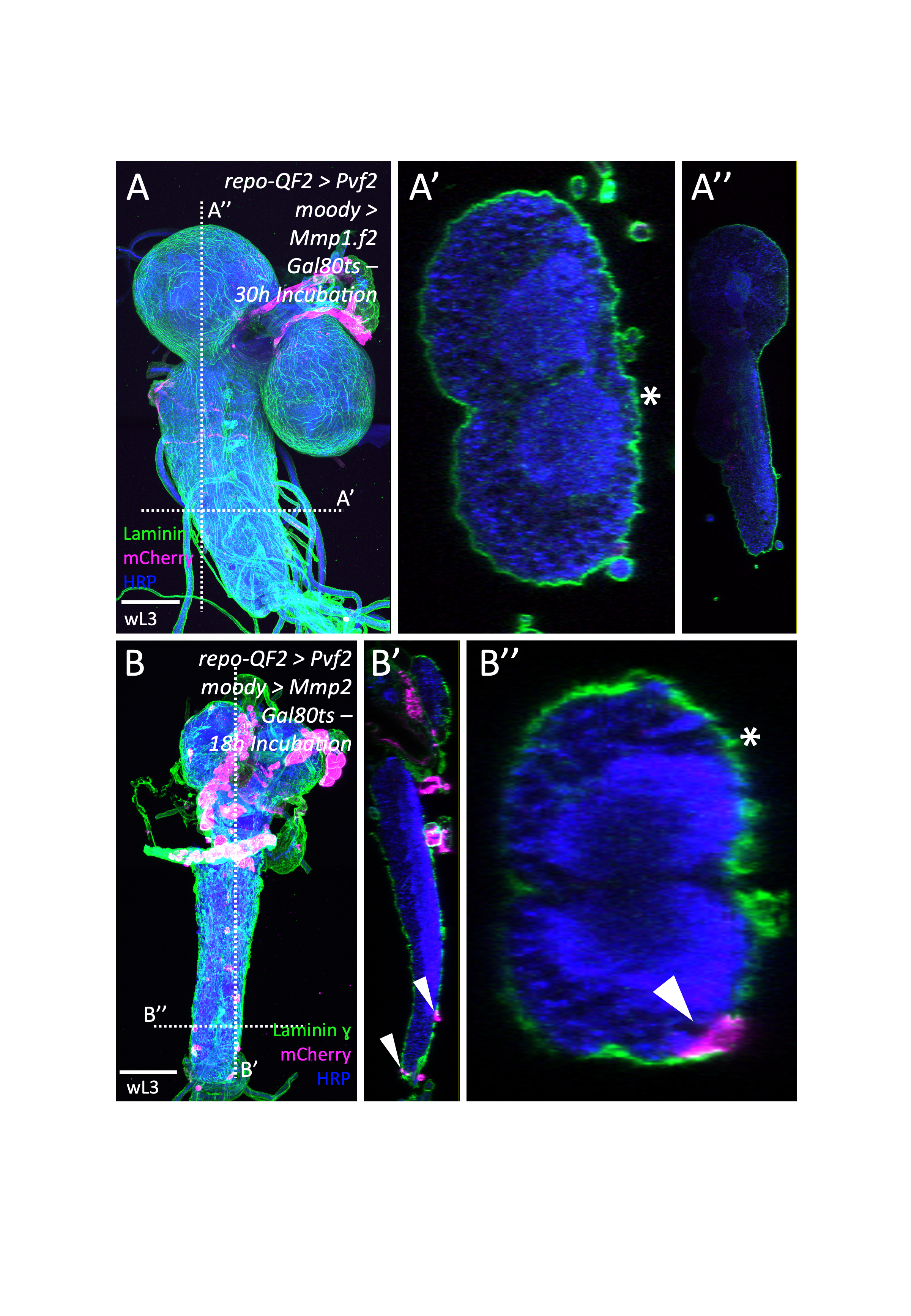

Supplement: S5 Fig — (A) Larval CNS with a panglial QUAS-directed Pvf2 expression and Gal4 induced Mmp1 expression in the subperineurial glia. Mmp1 expression was restricted to late larval stages by a ubiquitously expressed Gal80ts and an appropriate temperature regime. Brains were stained for Laminin-γ (green), mCherry expressing macrophages (magenta), and HRP (blue) to label neuronal membranes. The neural lamella appears rather intact, and macrophages never invaded the larval CNS (A′, asterisk). n = 19. Scale bar, 100 µm. (B) Upon expression of Mmp2 macrophages breach the neural lamella and are firmly attached to the CNS (B′, B″, arrowheads). The neural lamella appears disrupted (B″, asterisk). n = 5. Scale bar, 100 µm. (TIFF) [file pbio.3003035.s005.tiff]

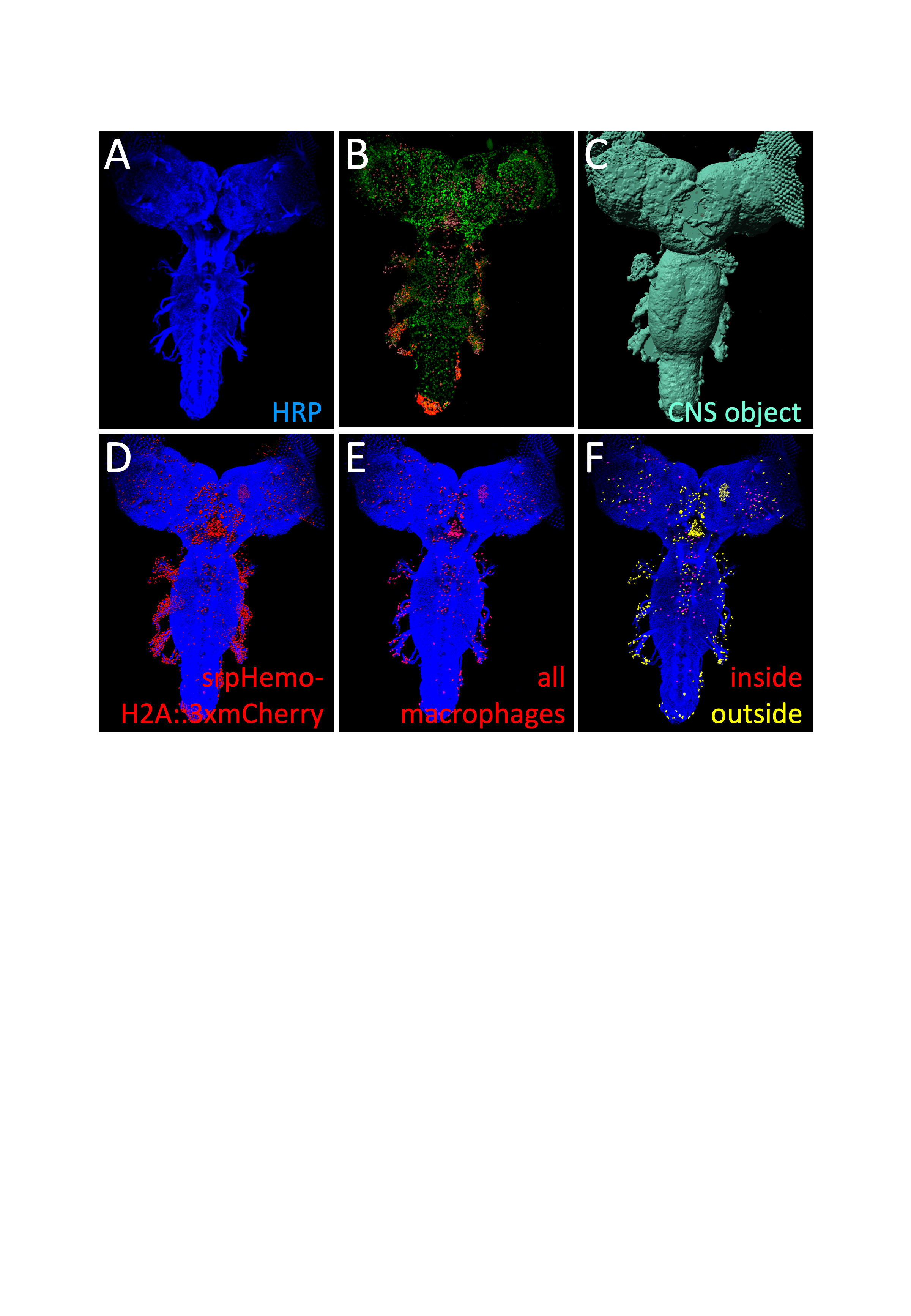

Supplement: S6 Fig — Quantification of infiltrated macrophages was performed with arivis Vision 4D. (A) The HRP staining of a pupal CNS used for segmenting the CNS. First, the HRP signal was denoised by discrete gaussian. (B) Same CNS, anti-Repo and anti dsRed staining to show glial nuclei (green) and macrophages (red). (C) In order to fill regions within the CNS with low-HRP staining signal, the CNS object was enlarged by region growing and later used for defining infiltrated macrophages by machine learning. (D) Objects expressing the nuclear macrophage marker srpHemo-H2A::3xmCherry were created using the blob finder. (E) These objects were filtered against co-localization with Repo (B) resulting in segmented macrophage nuclei. (F) Subsequently, these objects were classified with machine learning into macrophages inside (magenta) and outside (yellow) the CNS. (TIFF) [file pbio.3003035.s006.tiff]
