# Supplementary material for: Macrophage invasion into the Drosophila brain requires JAK/STAT-dependent MMP activation in the blood–brain barrier
Source: PLoS Biol. 2025 Feb 20;23(2):e3003035. doi: 10.1371/journal.pbio.3003035 (PMC11908702; doi:10.1371/journal.pbio.3003035)

Loading Plan for all samples

anti-dsRed						anti-tubulin					
			S1	S1	S1	S1	S1	S1			
			S2	S2	S2	S2	S2	S2			
S3	S3	S3	S5	S5	S5	S3	S3	S3	S5	S5	S5
S4	S4	S4	S6	S6	S6	S4	S4	S4	S6	S6	S6
S7	S7	S7	S9	S9	S9	S7	S7	S7	S9	S9	S9
S8	S8	S8	S10	S10	S10	S8	S8	S8	S10	S10	S10

Dot Blot control (*repo-Gal4 > nLacZ; srpHemo-H2A::3xmCherry*)

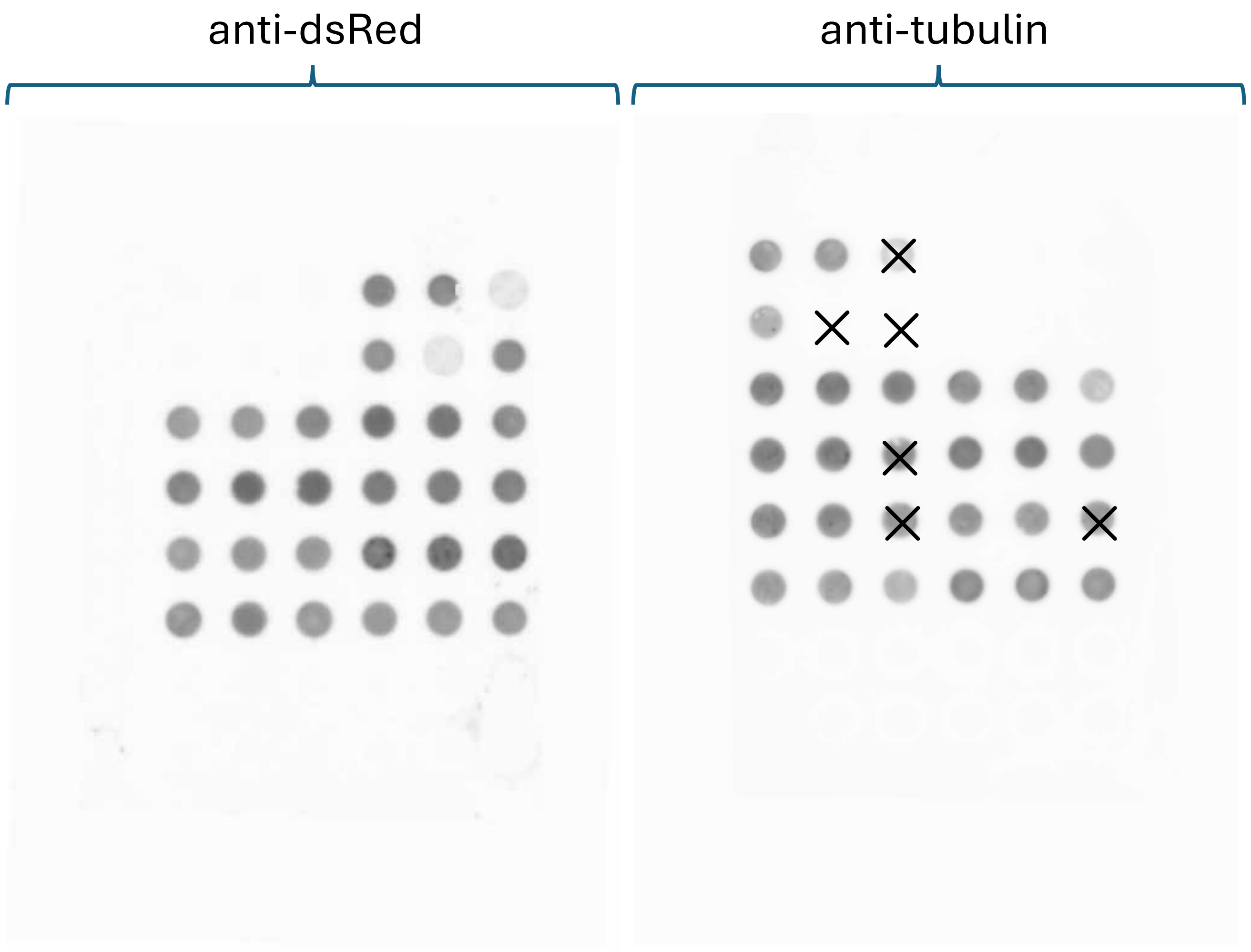

Dot Blot Immunity induction (*repo-Gal4 > PGRP-LE; srpHemo-H2A::3xmCherry*)

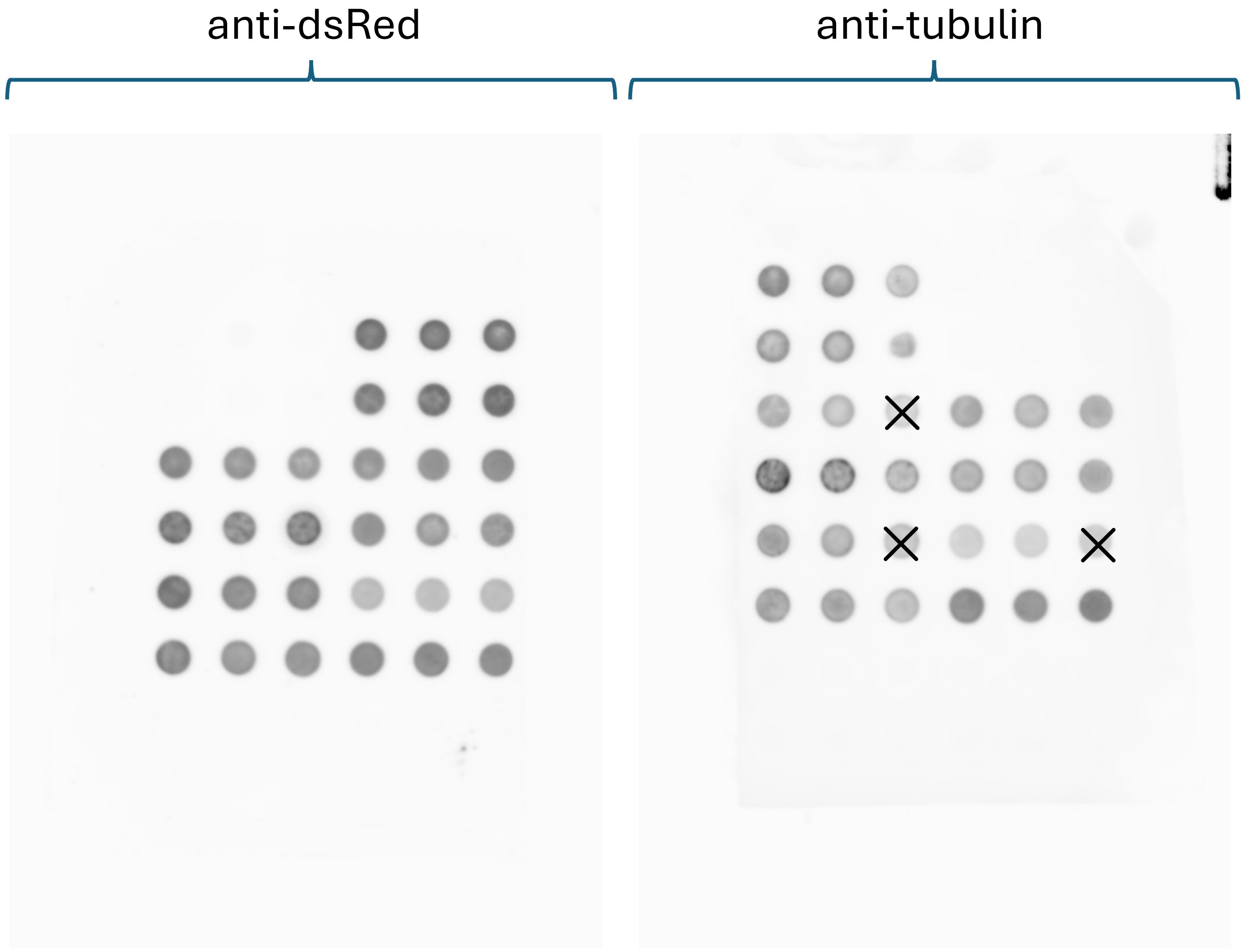

Dot Blot Immunity induction w/ STAT92E knockdown

(*repo-Gal4 > PGRP-LE, STAT92E*^{dsRNA}; *srpHemo-H2A::3xmCherry*)

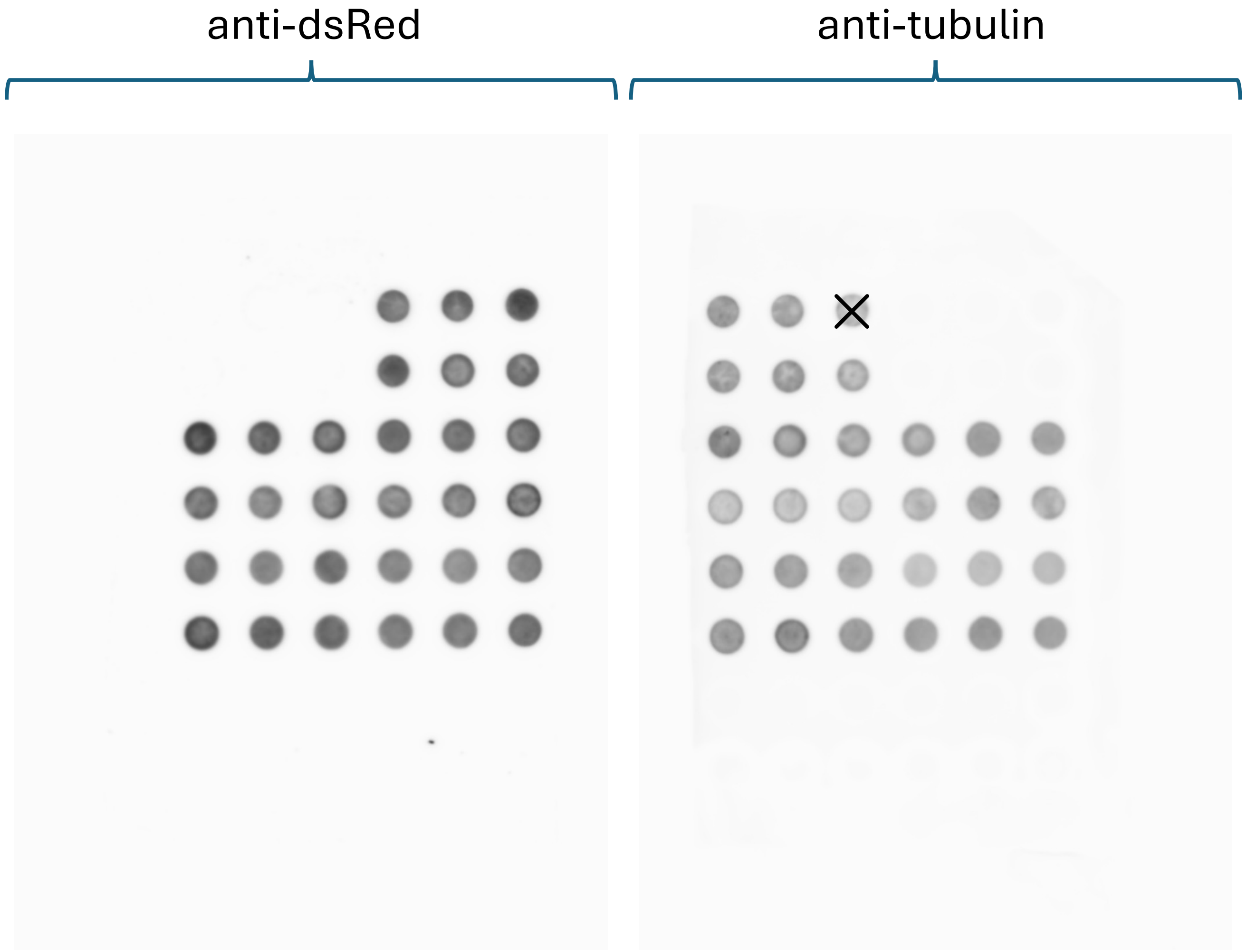

repo-Gal4 > nLacZ; srpHemo-H2A::3xmCherry
anti-dsRed

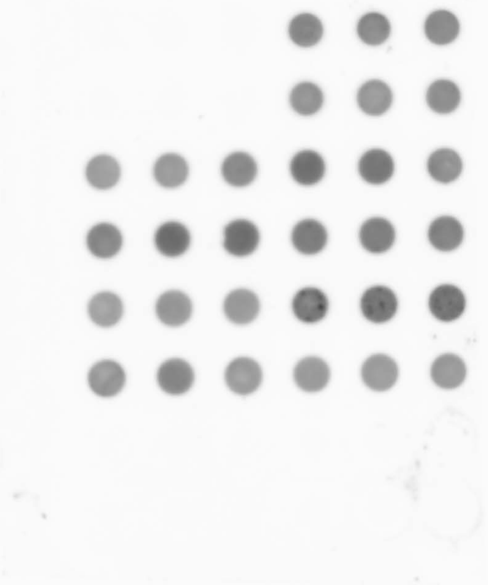

repo-Gal4 > nLacZ; srpHemo-H2A::3xmCherry

anti-tubulin

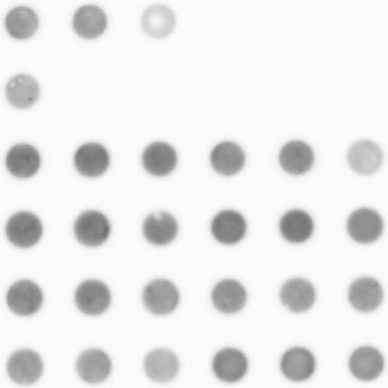

repo-Gal4 > PGRP-LE; srpHemo-H2A::3xmCherry

anti-dsRed

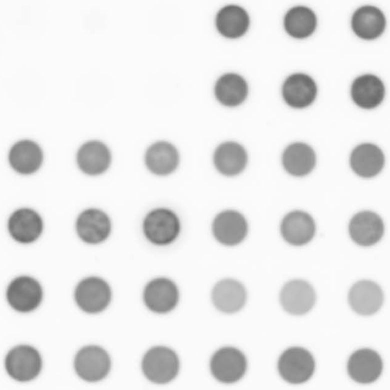

repo-Gal4 > PGRP-LE; srpHemo-H2A::3xmCherry

anti-tubulin

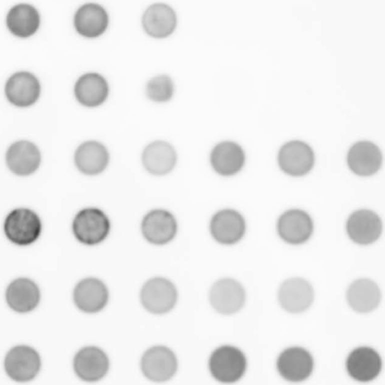

repo-Gal4 > PGRP-LE, STATdsRNA
srpHemo-H2A::3xmCherry

anti-dsRed

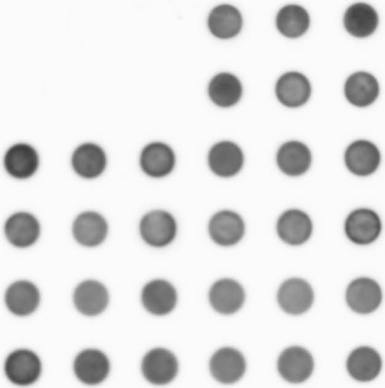

repo-Gal4 > PGRP-LE, STATdsGFP
srpHemo-H2A::3xmCherry

anti-tubulin

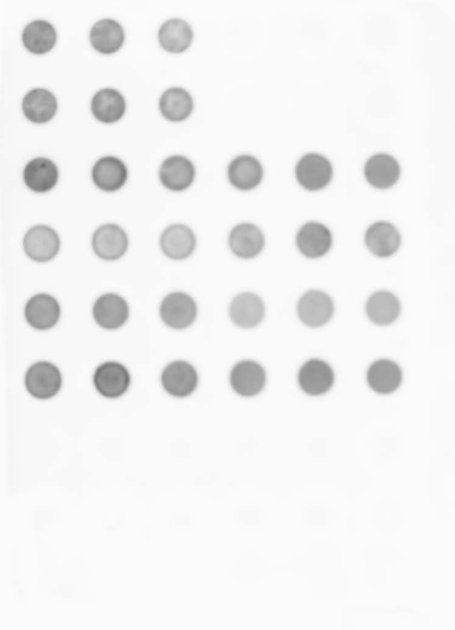

Supplement: S1 Raw Images — The loading scheme and six dot blots performed to quantify the number of macrophages in third instar larvae of the indicated genotypes are shown. Dot blots stained with the antibody indicated. Each sample (one larva) was applied as three technical replicates. Samples excluded from the analysis are indicated by a cross. (PDF) [file pbio.3003035.s007.pdf]
