# Supplementary material for: Macrophage invasion into the Drosophila brain requires JAK/STAT-dependent MMP activation in the blood–brain barrier
Source: PLoS Biol. 2025 Feb 20;23(2):e3003035. doi: 10.1371/journal.pbio.3003035 (PMC11908702; doi:10.1371/journal.pbio.3003035)
Supplement: Attachment — Submitted filename: rebuttal.pdf [file pbio.3003035.s018.pdf]

Dear Editor, dear reviewers,

We are thankful for all your comments and greatly appreciate the positive evaluation of our manuscript. We have now addressed all comments which we detail in the below point to point response. We put the text of original reviews in grey font, our responses are in black. We have to apologize that it took a bit longer to finish everything.

All the best

Christian

Reviewer #1:

… The manuscript is well-written and experiments are logical. The authors make use of the well-established glial-specific GAL4 to genetically manipulate IMD, MMP, and JAK/STAT signaling in a tissue-specific manner. They use immunohistochemistry of the larval brain to determine changes in neural lamina and macrophage invasion. The conclusions are generally well supported by the evidence. The only weakness is that the macrophage invasion quantification is not well defined and may need an additional control (see below for suggestions).

Specific Comments/suggestions:

1. The method of quantifying macrophage number is unclear as described.

We have detailed the method (arivis Vision4D) used for quantifying macrophage number and added this to materials and methods. We also added one additional supplementary Figure further demonstrating the procedure.

Is it possible that the genetic interventions of using dsSTAT/ and other genetic manipulations resulted in overall decrease in macrophage number surrounding the brain?

We are very thankful for this suggestion! The number of total macrophages is indeed an important figure. Several papers report on macrophage number, however, the numbers vary a lot, which is possibly due to technical issues (e.g. bleeding of the animal). For our experiments the exact number of macrophages is not relevant. Rather it is important to demonstrate that the STAT knockdown does not result in a general reduction in the number of macrophages and thus indirectly causes less infiltration into the CNS. To address the we took an indirect measure and performed dot-blot experiments where we assayed the amount of macrophage expressed mCherry. The method is detailed in Materials and methods section and the little graph shown on the left is now added to Figure 5. The result of these experiments is that the immunity induction slightly increases the number of macrophages, but concomitant knockdown of STAT92E does not significantly change the number of macrophages.

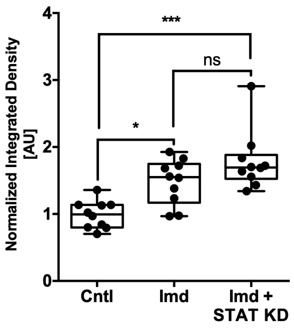

It would be helpful to have infiltrated macrophage numbers you presented as a ratio over total macrophages observed in your image (or number of macrophages in the hemolymph).

We have not changed the way to indicate the number of invaded macrophages. Normalizing this number to the overall available number of macrophages is difficult due to a number of reasons. For example, variable numbers of the macrophages are sessile and appear fixed in specific regions of the animal. In addition, the number of macrophages adhering to the CNS depend on the preparation method. Therefore, we think that the total number of infiltrated macrophages is the most relivable number.

2. Figure 6A image stitching looks to be abnormal.

We are very sorry but this effect is due to our imaging setup. Our microscope (LSM 880, Zeiss) allows automatic image acquisition and subsequent stitching. Since we always want to see the full brain hemispheres the stitching always occurs in the thoracic neuromers of the ventral nerve cord.

Below are three additional examples all showing Mmp1 localization in third instar larval brains with the same genotype as shown in Figure 6A. It is a technical limitation of the microscope.

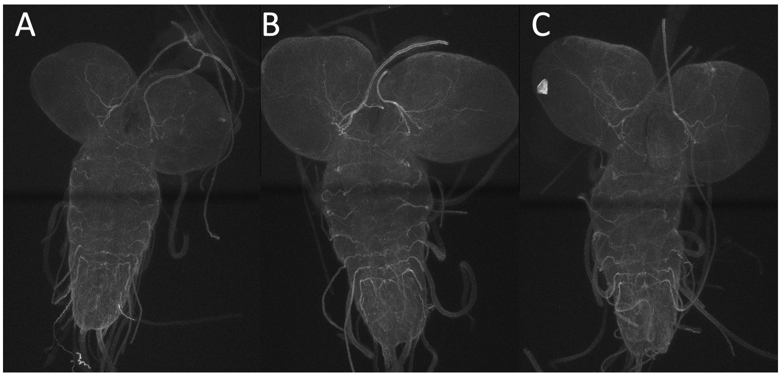

3. Number of experimental replicates are not mentioned.

We apologize for this and have added this information now to all Figures legends!

4. n's are missing in Figure 1.

See above comment

5. How many larval brains were used for the qRT-PCR experiments?

RNA was isolated from 15-17 third instar larval or 7-10 pupal brains for each of the genotypes. This information is now included on Materials and Methods.

6. Could the authors clarify the reasoning behind using 2^-dCT rather than the typical representation as fold change over biological control (2^-ddCT)?

Since we are also looking at expression in several different stages, such a representation would eliminate relative change in expression strength. Since the control is indicated, both the fold change over biological control as well as the relative expression levels during development can be followed on the figure.

7. Figure 4D - STAT::GFP expression does not appear to overlap with mdr65 expressing cells at the center of the VNC (line of small circles in the middle). Could you speculate on why this is?

We are sorry that the old Figure did not show this properly. We included now a better image where we stained the neural lamella and also changed the text:

(77). Co-labeling experiments demonstrated expression of the STAT reporter in both glial cell types**(Fig. 4D)**. STAT::GFP is activated in the subperineurial glia which projects fine processes into the CNS cortex as well into the perineurial glia layer (Figure 4D'-''', arrowhead). The perineurial glial cells also activate the STAT reporter (Figure 4D'-D''', arrow). Thus, both cell types of the BBB can activate JAK/STAT signaling.

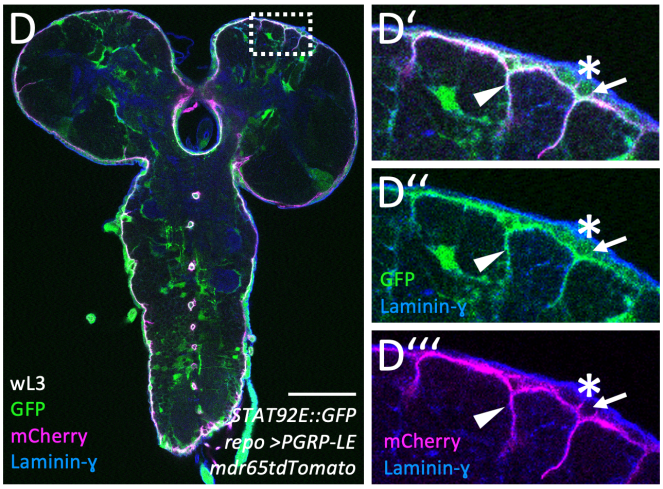

Above is the new part of Figure 4 showing STAT92E activity in subperineurial and perineurial glial cells of the blood-brain barrier.

8. Line 46-48: while endothelial cells do produce components of the basement membrane, it might be more balanced to mention the involvement of pericytes as well (https://doi.org/10.1038/srep36450; https://doi.org/10.3390/cells11101707).

We are thankful for the suggestion and added the references.

9. A few discussion and conclusion points could be clarified - the authors mention that the infiltration of immune cells can be a physiological or pathological change, however the idea is very general. My question is, in your model, until what point would an infiltration of immune cells associated with inflammation be considered necessary for physiological processes and at what point would it be considered pathological?

Bacterial infection can occur in nature, but the number of invading macrophages is very low (1-2 macrophages, see [Winkler, 2021 #33]). Since we usually deal with invasion of >50 macrophages, we consider it pathological. We added corresponding statement right at the beginning of the discussion.

10. Does the infiltration of immune cells associated with a neuroinflammatory event during the pupal stage of Drosophila impact the development of the fly or adult behavior and neurological phenotypes?

Yes, the number of infiltrated macrophages has strong implications. As higher the number as lower is longevity and fitness. We have analyzed these aspects in a previous study [Winkler, 2021 #33] and thus have not further commented on this finding.

Reviewer #2: …This manuscript is well written, but requires some clarification to non-specialists, especially to those not well versed in Drosophila genetics (please see major concern #1). The experiments are logical and the data support the claims by the authors, although some control experiments are needed for some of the results. Overall, this reviewer finds the study very interesting and significant given that neuroinflammation plays a prominent role in many neurological diseases. While the applicability from invertebrates to vertebrates may not be relevant for all of the results presented, the manuscript provides valuable information that could be translatable to human studies.

Major concerns:

1) This reviewer found the genetic lines used very difficult to understand initially. For example, in several sections of the results, it was challenging to follow the experiments being performed. This reviewer suggests providing a detailed description of the lines used and the rationale behind each line so that the reader does not have to spend too much time trying to figure out what was done. For example, repo-Gal4 was not described anywhere in the manuscript. To determine that this line expresses in glial cells, this reviewer had to refer to Winkler et al Sci Adv 2021. While some of this information is provided in the legends, a description in the main text would have been very useful. Perhaps a figure with an overview of the experimental lines and structures as shown in Winkler et al Sci Adv 2021 could help clarify these important points.

We are thankful for this advice and have added an explanatory Figure in the supplement and link to this figure directly at the start of the results section.

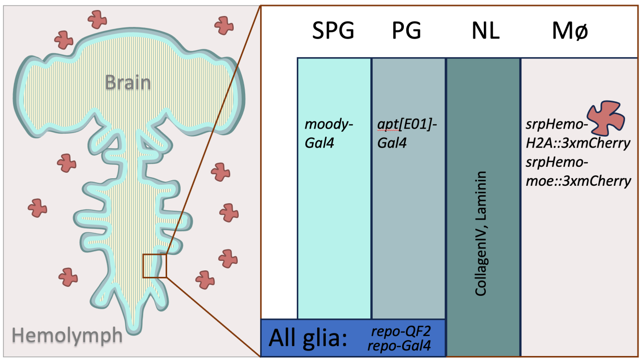

NEW **Figure S1**: Schematic representation of all main tools used in this study. *repo-Gal4* and *repo-QF2* direct expression in all glial cells. moody-Gal4 directs expression in the subperineurial glia (SPG), apt[E01]-Gal4 directs expression in the perineurial glia (PG). The neural lamella (NL) can be labelled using a GFP protein-trap insertion in the *collagen IV* gene or by staining using anti-Laminin-γ. Macrophages (Mø) can be visualized using *srpHemo-H2A::3xmCherry* or by *srpHemo-moe::3xmCherry.*

2) Knockdown of STAT, dome, and hop in results section "JAK/STAT signaling is required during macrophage invasion" shows a dramatic decrease in the number of infiltrating macrophages. However, this data does not distinguish between reduced infiltration versus a reduced total number of macrophages. This could be demonstrated by showing that the number of macrophages in the periphery is not impacted by knockdown of one or more of these genes.

We absolutely agree to this comment and added quantification of the number of macrophages in the different conditions, please see response to reviewer 1.

3) The analysis of upd gene expression was performed on CNS preparations. However, it seems likely that immunity induction could also regulate expression outside of the CNS, impacting BBB permeability and macrophage infiltration. Along these lines, the study used upd2 and upd3 knockouts as opposed to glia-specific dsRNA for knockdown. With dsRNA, the CNS effects of upd1 could potentially be examined as well. Ultimately, the question is: do Unpaired cytokines translocate across the Drosophila BBB and is this transport bidirectional? For example, the author's model (Fig. 10) shows the possibility of Pvf2 moving across the BBB.

This is a very good suggestion. We have now performed the requested RNAi based knockdown experiments. For upd3 we obtained a significant decrease in the number of infiltrated macrophages upon panglial RNAi based knockdown using two independent dsRNA expressing lines. For upd1 two independent dsRNA knockdown lines are available and again both lines caused a reduction in the number of invading macrophages, however, for one of the lines this reduction is statistical non-significant. For upd2, we had three independent dsRNA expressing lines. For one of these lines we noted a highly significant reduction in the number of invading macrophages. For the two remaining lines we found an increase that according to Kruskal-Wallis is statistically not significant.

To address the question whether Unpaired cytokines or Pvf2 can translocate across the Drosophila BBB we conducted a large number of experiments. We performed split-GFP type and trans-tango like experiments. We expressed human glucagon, which has a similar molecular weight as Pvf2/Upd within the CNS and co-expressed the glucagon receptor in the perineurial glia. Unfortunately, none of these experiments yielded any conclusive results.

Additional comments:

1) In Figure 2, Mmp1 staining is shown to be localized to the Drosophila BBB. However, there are no co-localized markers demonstrating that this staining represents the BBB (i.e. what cell type(s) show that this structure is the BBB as shown in Fig 4 for example?). Without significant familiarity with the Drosophila BBB, this information would be useful to put the results in context.

We added new images to show colocalization at the Drosophila BBB similar to what is shown in Figure 4. In addition, we also added now images showing the localization of Mmp2.

2) Figure 3A and 3B are lacking controls for "No Immunity Induction." This would be useful to compare to TIMP and MmpdsRNA. In other words, does TIMP restore macrophages to "no immunity induction" levels and does MmpdsRNA reduce macrophages from baseline? Also, HRP is not described (Assuming that this is neuronal membrane as described in Figure 1 legend?).

The control with “No Immunity Induction” has no invading macrophages, and therefore we did not include it in the graph. So neither TIMP expression nor Mmp knockdown completely blocks invasion of macrophages triggered by immunity induction. We added the missing description of HRP in the legend of Figure 3.

3) In Figure S2A, CTCF is not defined. According to the text, it could be assumed that this is the level of puc::GFP expression, but is not described in the figure legend.

We have defined CTCF in Material and Methods and now reference this in the Figure legends

Furthermore, it would be relevant to provide images of puc::GFP expression to demonstrate that localization of the signal does not change upon IMD. Likewise,

knockdown of basket appears to unexpectedly upregulate Mmp1 compared to control (Fig. S2B,C), although this data is not quantified.

We have added the images as requested. We apologize for the bad selection of exemplary images showing Mmp1 expression. From our data we did not have the impression that Mmp1 expression changes in response to basket knockdown.

4) Please explain how STAT92E being downregulated. For example, in Fig. 5A, is STATdsRNA actually UAS::STAT92EdsRNA? This is not described in the text or methods or at least not easily found.

Yes, we used *UAS-STAT92EdsRNA*. We are sorry for the sloppy description. STAT is lab slang and we made sure that in all instances we now correctly call the Drosophila gene STAT92E in the text and in the Figures.

5) The JAK/STAT signaling seems to contribute to the level of Mmp expression (Fig. 6). However, these data do not show statistical significance. As n=4 for this analysis and there appears to be a wide range of expression between data points, perhaps increasing the numbers would provide significance. This also seems to be the case for expression of the upd genes (Fig. 7). Please elaborate as to why more samples were not included in these analyses.

We are thankful for this advice, we have increased the number of analyzed brains and included them in the statistical analysis, which leads to significance in most cases.

6) There are several concepts throughout the manuscript that cite review papers rather than the primary literature. Please consider modifying the references accordingly.

We carefully went through the manuscript and reduced / changed the citations accordingly.

Reviewer #3: This manuscript by Winkler et al attempts to unravel the signalling pathways driving immune cell invasion into the brain during neuroinflammation using Drosophila as a model system. The manuscript follows up from previous work from the lab which established a neuroinflammation model in drosophila to study macrophage invasion into brain. The current work identifies a role for unpaired

cytokines in activating JAK/STAT signalling pathway to drive changes in ECM remodelling allowing macrophage invasion. The identification of the molecular pathway is an advance on the previous work and demonstrates interesting parallels between the vertebrate and invertebrate neuroinflammatory pathways. A limitation of the work is that while the players are identified, the mechanistic detail of how the pathways work is not present which makes the manuscript rather descriptive. It is not clear how the two MMPs are differentially activated (is there a cell type specific requirement?).
Also, the evidence supporting the differential expression of JAK/STAT signalling in the two cell types is rather weak.

1. The structure of the brain in the pupal stages look different in the inflammation model as compared to the control. Is there a structural degradation of the brain ? Does that happen before the loss of the ECM? The authors could supply transmitted light images to make this clearer.
You are absolutely correct. The shape of the CNS is slightly abnormal which is likely due to the increased expression of MMPs which occurs before ECM remodeling is detectable. We had previously reported that glial expression of Mmp1 results in lethality whereas glial expression of Mmp2 leads shape changes of the CNS. We have added this information to the text.

2. The authors do not discuss in any detail why the MMP expression patterns are quite temporally different. The levels of MMP1 in the inflammation model is high in the larval stages and stays up till 8APF and then drastically goes down. The MMP2 levels only increase at 8h APF but and then goes down. Interestingly the Laminin levels go down at 8 h APF and then consistently stays down. It would be interesting to understand why the ECM remodelling is temporally different from the MMP expression and what contributes to the differential MMP expression. Interestingly, loss of both the MMPs using RNAi has the same phenotype on Laminin. Also, can the authors use an antibody against MMP2 to see the expression pattern ?
This is an important question and we had missed in the first version of the manuscript. We now added the following to the discussion:
Interestingly, the two genes encoding MMPs in Drosophila show differential regulation upon immunity induction. This may be due to the differential functions of

Mmp1/2. While Mmp1 is primarily a collagenase but also targets membrane bound receptors, Mmp2 can act as a gelatinase. For example, during fat body remodeling during metamorphosis, Mmp1 preferentially cleaves DE-cadherin junctions between individual fat cells, while Mmp2 preferentially degrades the surrounding ECM (Jia et al., 2014). Thus, both MMPs likely act in a sequential and cooperative manner.

3. A technical concern within the manuscript is the interchangeable use of both the larval brains and the pupal stages for different analysis. For example, the STAT reporter expression is only analysed in the WL3 stages and not the pupal stages. Does the STAT signalling go down later, leading to a lower level of MMP induction?
We have indicated which developmental stage was used in the paper. We had determined STAT92E activity in younger pupal brains (Fig. S3) and have now analyzed one day old pupae. Here, we found no STAT92E activity in control and immunity induction. The data are shown below but are not added to the manuscript. We do, however, mention that no STAT92E activity is detected in 24h APF pupae.

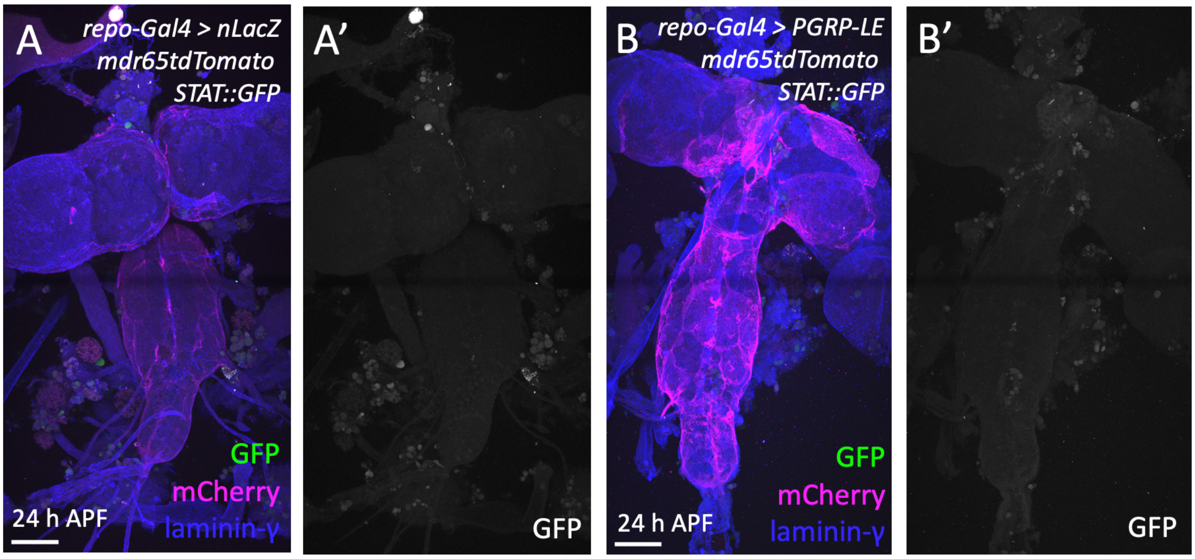

STAT92E activity in control (A) and immunity induction pupae (B). No STAT92E activity can be detected.

4. The evidence of differential STAT reporter expression in the two glial cell types is weak. From the images in Figure 4D, it is not clear how the authors are distinguishing the two cell types. The images provided in D'-D''' look like a side view with the STAT reporter expressed on both the apical membranes and the cortex of the same cell which expresses Ds-red. The authors should distinguish the two cell types in the images to provide evidence of differential expression.

We are thankful for this comment and have redone this analysis and changed the Figure. Please see comments to reviewer 1.

5. A major concern within the manuscript is the lack of information surrounding the automated workflow used to quantify the invaded macrophages. The authors should provide more details on how this was done, how was the macrophage assigned as invaded. Would the macrophages on the surface be counted? Images of the invaded macrophages could be added to the supplementary data.
We have added a detailed description of the pipeline used for macrophage counting and described the protocol used for the arivis 4D software. See also comment above.

Technical concerns:
1. The authors should provide cartoons to better indicate the different areas of the brain and the cells types to readers who are not familiar with the field.
We added a new supplementary Figure to address this and the concern of reviewer 2 regarding the different genetic tools used in the study.

2. There is no quantification of the laminin expression and since that is used as the major readout of ECM remodelling, addition of this quantification would be essential to provide a more quantitative description of the ECM levels. For example, calculating the fluorescence intensity for Laminin::GFP expression in figure 1 under wild type and under induced immunity conditions would provide clearer evidence that Laminin::GFP expression reduces over time and that this process is quickened when an immune response is induced. This same principle can be applied to figures 2, 3 and 4.
We wish we could do this quantification in a reliable way as this is a technically challenging dissection method, the presence of other tissue (fragments) which neither can be physically removed (this would destroy the lamella) and nor be removed in an automated segmentation (which we tried), quantification numbers would blend out the actual biological image. For the purpose of this study, a quantification of ECM degradation would be nice but in our view is neither necessary nor possible. However, we increased the n for each genotype. The data shown below was stained and recorded with the exact same settings. While in control brains a smooth surface of with a Laminin-γ is observed at 8 h APF, brains with a panglial

immunity induction show a partially fragmented or accumulated Laminin-γ staining. Moreover, those images show fragments of other tissues around as well as on top of the nervous systems, which could not be removed during dissections.

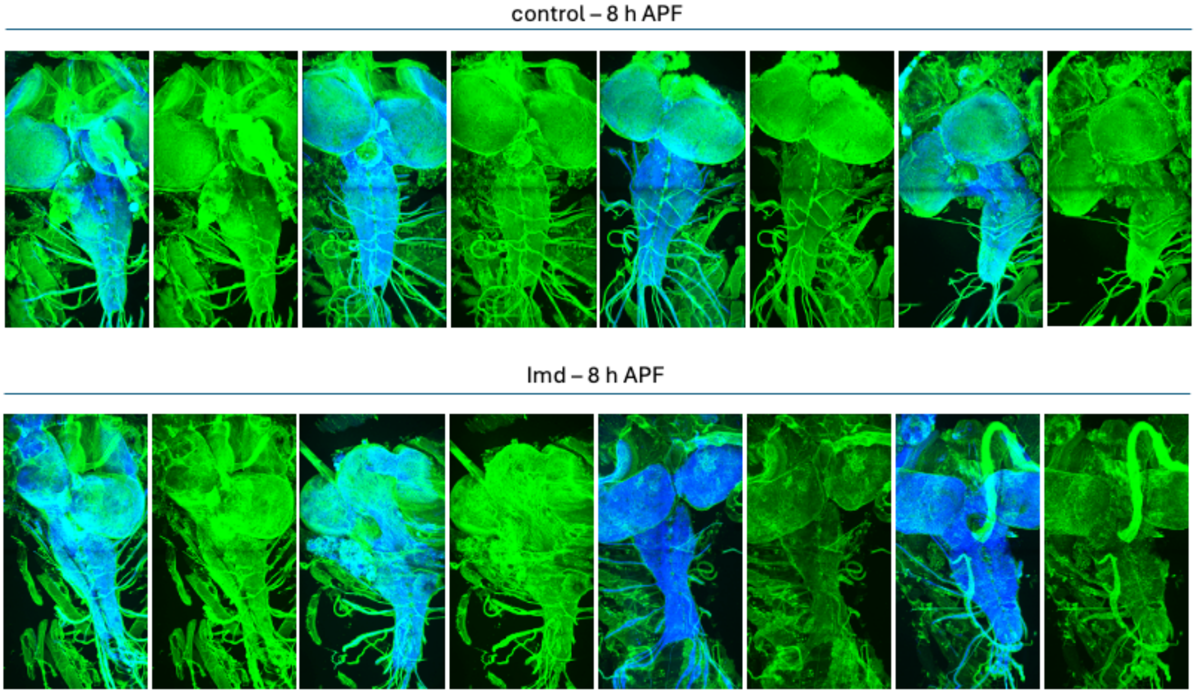

Maximum intensity projections of filet preparations of 8h APF old controls (*repo-Gal4 > nLacZ*) (top panel) and panglial immunity induction (*repo-Gal4 > UAS-PGRP-LE*) (lower panel). All filet preparations were stained with Laminin-γ (green) to label the neural lamella and HRP (blue) to label neuronal membranes. Moreover, all filets were recorded with the exact same image settings.

3. The authors describe that the expression of GFP tagged Collagen affects Laminin levels. This is a surprising finding and while the use of Laminin as a proxy for ECM remodelling is fine, it would be interesting to understand why the expression of the Collagen transgene has this effect.

We agree that this is an interesting observation. Possibly, the CollagenIV::GFP fusion protein affects the supramolecular structure of the matrix networks but how this is mediated must be resolved in a future study.

4. Are the dsRNAi used in the manuscript (for the MMPs) previously described? It would be good if the authors can use more than one RNAi line for the proteins to negate any off-target effects.

All dsRNA expressing fly stocks were obtained from the Bloomington stock center as indicated and for all these lines many publications are listed (up to 24 references). We have now repeated the dsRNA experiments with independent dsRNA lines and obtained the same data as given in the manuscript for *Mmp1*, *hop* and *STAT92E*. For *dome* we could use only one dsRNA line (used in 14 other publications) due to chromosomal location problems. For *Mmp2* there are only two dsRNA lines available in Bloomington. One of those has been cited only once, whereas the other dsRNA line targeting Mmp2, which we used in our study has been cited 8 times. We tested now the second line and it appears unfunctional.

Minor concerns:

1. The signalling pathways described from page 5, line 94 could be clearer to the reader if presented with a schematic diagram.

We have added a new schematic including the most relevant genes in Figure 10.

2. The CollagenIV::GFP data in supplementary figure 1 could be moved to figure 1 as this is a key point describing why the authors focus on laminin throughout the paper.

We are thankful for this suggestion and changed the Figure accordingly

3. In figure 3, a figure depicting what the authors defined as successful macrophage invasion would help the reader visualise where the macrophages are invading. A schematic accompanied with a microscopy image would work well.

We have now added an example image to the description of the arivis4D based quantification pipeline.

4. In figure 6, the axis for the graphs in C and D differ from those in figure 2 making it difficult for the reader to compare the two.

We are sorry, have fixed this now.

5. Some of the figure legends lacked detail. The legend of figure 1 contains details that could be made simpler.

We have carefully revised the Figure legends.

6. One of the panels in supplementary figure 5 looks out of focus making it difficult to compare it to its control.

We are sorry for this impression. Figure S5B'' shows an orthogonal section of the stack shown in B. The level of image resolution does not allow better image quality. The important point conveyed by the image is that macrophages move under the ECM even in larval stages. Please also note the elongated shape of the CNS (see comments above).

7. 'Pupariation' is spelt incorrectly in the figure legend for figure 1.

Changed-.

8. In figure 4, the image titles are overlapping with the CNS images.

We have changed this
